# Oxygen diffusion kinetics, buffering capacity and phenotype: A narrative review of oxygen physiology and later phenotype in very preterm infants

**DOI:** 10.1113/EP093607

**Published:** 2026-04-07

**Authors:** Chad C Andersen, Danielle N Bailey, Tara M Crawford, Michael J Stark

**Affiliations:** ^1^ Robinson Research Institute Adelaide University North Adelaide SA Australia; ^2^ Department of Neonatology Women's and Children's Hospital North Adelaide SA Australia

**Keywords:** buffering capacity, cerebral palsy, Fick's law, haemoglobin, near infrared spectroscopy, oxygen, oxygen consumption, oxygen delivery, retinopathy of prematurity, transfusion

## Abstract

Clinicians often view exposure to supplementary oxygen in preterm infants as a simple reciprocal trade‐off between mortality and the risk of vision‐threatening retinopathy, but this perspective oversimplifies the underlying physiology. Oxygen moves through a series of spatially distinct compartments without intrinsic regulation, with Fick's law governing the entire process. We suggest that retinopathy of prematurity and cerebral palsy represent opposite ends of a shared continuum of oxygen diffusion injury. Several physiological concepts define this risk matrix. The *spatial critical threshold* indicates the structural limits of immature microvasculature that impact the diffusion radius. The *extinction gradient* marks the point at which the capillary‐to‐cell gradient becomes flat, leading to flux collapse and intracellular hypoxia. Conversely, the *hyperoxic injury threshold* identifies the point at which intracellular oxygen tension becomes harmful. Lastly, *buffering capacity* refers to the oxygen bound to haemoglobin in the venous circulation, downstream of metabolism, which is available to buffer temporary mismatches between delivery and consumption. These thresholds explain how hyperoxia and hypoxia can coexist within the same capillary, clarifying distinct clinical phenotypes. Framing oxygen injury this way clarifies contradictions in neonatal trials and offers a physiological model relevant to other diffusion‐limited conditions.

## Abstract (highlights)

- **What is the topic of this review?** This review examines the relationship between oxygen physiology and later phenotype in very preterm infants.
- **What advances does it highlight?** This review proposes that retinopathy of prematurity and cerebral palsy lie along a continuum of oxygen diffusion injury, with phenotype determined by the interaction between diffusion gradients and buffering capacity. It distinguishes the effect of population‐based oxygen saturation targets from those of individual physiologic exposure.

## 1 INTRODUCTION

Preterm birth imposes an immediate physiological challenge to the newly born preterm infant. Positive pressure ventilation adversely affects blood flow while clinicians allow haemoglobin to fall significantly, thereby altering oxygen carrying capacity and systemic oxygen delivery ($D_{O_{2}}$) (Kirpalani et al., 2020; Kluckow & Evans, 2000). Concurrently, oxygen consumption (${\dot{V}}_{O_{2}}$) increases dramatically, particularly due to the combined metabolic cost of thermal homeostasis and parenchymal lung disease (Adamson et al., 1965; Bauer et al., 1998; Chessex et al., 1981; Sauer et al., 1984; Schulze et al., 2001; Weinstein & Oh, 1981). Together, this combination destabilises oxygen kinetics and substantially reduces the capacity to buffer against a mismatch between ${\dot{V}}_{O_{2}}$ and delivery. Moreover, the preterm infant must respond with constituent parts better suited to an intrauterine environment (Delivoria‐Papadopoulos et al., 1976; Oski, 1971).

Typical approaches treat exposure to supplemental oxygen as a binary choice between vision‐threatening retinopathy and mortality or neurosensory disability (Askie et al., 2018). Moreover, expert opinion suggests that institutions should choose a population target range based on local rates of important outcomes (Schmidt & Kirpalani, 2022; Schmidt & Whyte, 2020). Yet according to Fick's law, oxygen flux depends on the concentration gradient and spatial geometry (Lumb & Thomas, 2020) while buffering capacity determines the temporal duration of normoxic conditions. Together, these three factors define a continuum of risk rather than a binary choice.

We propose that oxygen‐related injury in very preterm infants reflects constraints imposed by structural immaturity, diffusion geometry and limited buffering capacity on the local balance between oxygen delivery and consumption, with tissue‐specific consequences for organs such as the retina and periventricular white matter. These constraints give rise to characteristic patterns of injury.

This article is a narrative review that integrates established principles of oxygen diffusion physiology with contemporary neonatal clinical trial data to examine these mechanisms in very preterm infants. Rather than systematically re‐analysing outcomes, we focus on physiological processes that have been pivotal to current understanding and use this perspective to identify unresolved questions and directions for future physiological measurement.

## 2 HISTORICAL CONTEXT

Understanding how oxygen exposure affects preterm infants has advanced gradually, shaped by a series of small but important clinical and physiological steps. Early work by Haldane and Barcroft established core principles of oxygen transport and the consequences of inadequate supply, forming the basis for later neonatal practice (Barcroft, 1920; Haldane, 1917). Remarkably, they conducted several of these experiments on themselves.

In the 1940s, incubators capable of supplying sustained, high levels of inspired oxygen improved survival rates in low‐birth‐weight infants but also caused an *epidemic* of retrolental fibroplasia (RLF). The National Cooperative Study, the first significant effort to regulate oxygen exposure, randomly assigned infants weighing less than 1500 g to either routine oxygen ($F_{iO_{2}}$ > 50% for 28 days) or reduced oxygen. Mortality rates were similar (22% vs. 21%), but the occurrence of blinding eye disease was halved with reduced exposure, establishing a link between excessive oxygen and RLF (Kinsey, 1956; Kinsey & Hemphill, 1955). This prompted a period of restricted oxygen use in the following decades. Cross later estimated that there were 16 deaths for every case of blindness prevented (Cross, 1973).

The development of a standardised retinopathy of prematurity (ROP) classification in the 1980s enabled more targeted intervention trials. CRYO‐ROP showed that ablative cryotherapy for threshold disease halved the risk of poor retinal outcomes, establishing ROP as a treatable condition and reshaping management strategies (Palmer, 1990). Moreover, this study has methodological importance, as researchers randomised the eye, not the infant, with the other eye allocated to the alternative treatment (no treatment (control) or cryotherapy (intervention)).

The Stop Retinopathy of Prematurity (ROP) trial (The STOP‐ROP Multicenter Study Group 2000) investigated whether higher oxygen saturation targets (96–99%) in infants with pre‐threshold ROP (Stage 3 ROP) affected disease progression. Higher targets did not worsen overall progression (48% vs. 41%), but they increased pulmonary morbidity and appeared more effective in infants with plus disease (tortuosity and dilation of retinal vessels) (The STOP‐ROP Multicenter Study Group, 2000). Interestingly, in a subgroup of infants with plus disease, the treatment was more effective. These findings suggest that oxygen's effects depend on timing, vascular maturity and disease stage.

Attention then shifted to early postnatal life. Benefits of Oxygen Saturation Targeting (BOOST I) randomly assigned infants <30 weeks to either a standard (90–94%) or a high (95–98%) oxygen target until term, finding no survival benefit with higher targets but an increased risk of chronic lung disease (Askie et al., 2003). Importantly, this trial showed that masked oximetry‐based interventions were feasible, enabling subsequent international trials of larger sample sizes (Askie et al., 2003).

This led to the Neonatal Oxygenation Prospective Meta‐analysis Collaboration (NeOProM) (Askie et al., 2018) which pooled data from five conceptually similar trials (Benefits of Oxygen Saturation Targeting II (BOOST II United Kingdom, Australia and New Zealand) (BOOST 2013), Surfactant, Positive Pressure, and Oxygenation Randomized Trial (SUPPORT (USA) (SUPPORT 2010) and Canadian Oxygen Trial (COT)) (Schmidt et al., 2013) comparing lower (8%–89%) versus higher (91%–95%) saturation targets. NeOProM quantified the competitive tension between ROP and mortality (Askie et al., 2018). These trials clarified the effects of population‐based saturation targets on mortality and major morbidities and were consistent with the historical literature.

As monitoring technology advanced, the focus shifted downstream from the arterial to the venous compartment. Bale, Elwell and Tachtsidis traced the evolution from Jöbsis's early optical chromophore measurements to modern broadband or multiwave NIRS capable of detecting the redox state of cytochrome c oxidase (Bale et al., 2016), enabling assessment of tissue‐level oxygen sufficiency rather than relying solely on arterial metrics.

## 3 PHYSIOLOGY

Oxygen descends a staircase of pressure gradients across spatially distinct compartments according to Fick's law (Lumb & Thomas, 2020). The rate of diffusion depends on the surface area (radial, axial and longitudinal distances) and the steepness of the gradient between the capillary, the neighbouring cell and the index cell. Additionally, oxygen carrying capacity and blood flow determine the temporal adequacy of aerobic conditions.

Microvascular immaturity creates structural limitations in preterm infants (Wright et al., 2019). The sparse, irregular capillary network reduces diffusion surface area and increases radial distance, thereby heightening oxygen gradients. This establishes a critical spatial (radial, axial and longitudinal) threshold as the maximum distance or radius over which effective diffusion can maintain adequate intracellular oxygen levels.

**FIGURE 1:**
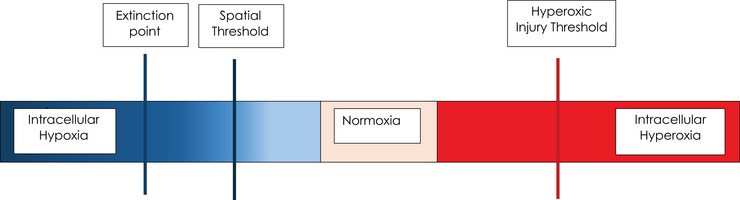
Oxygen sufficiency exists within a narrow range between hypoxic and hyperoxic injury. On the left side of the figure, hypoxic injury and the risk of cerebral palsy occur once the spatial distance threshold is exceeded or the capillary‐to‐cell gradient collapses (extinction point), or both happen together. On the right side of the figure, hyperoxic injury and the risk of retinopathy of prematurity develop once the hyperoxic threshold is exceeded, leading to steep gradients that drive excessive intracellular oxygen tension.

The gradients between capillaries and cells, as well as between individual cells, are the main adjustable factors in diffusion physiology. Without intrinsic control of the diffusion flux, the gradient can cause both intracellular hypoxia and hyperoxia, creating a dynamic threshold. As capillary partial pressure of oxygen ($P_{O_{2}}$) decreases along the vessel, the gradient progressively flattens. At the *critical extinction point*, the diffusion flux falls below the level required to sustain aerobic metabolism, causing intracellular $P_{O_{2}}$ to drop sharply, activating anaerobic metabolism and lactate buildup. Conversely, steeper gradients at the arterial end can raise intracellular $P_{O_{2}}$ above the upper limits of typical normoxic levels. Beyond this *hyperoxic or oxidative threshold*, cells experience oxidative stress, which can lead to damage. Therefore, proximal capillary regions are prone to hyperaemia or oxidative injury, while distal regions approach the extinction threshold and hypoxaemia (Figure 1).

The adequacy of oxygen over time (duration) depends on carrying capacity and blood flow. Mathematically, arterial oxygen content is determined by haemoglobin concentration and haemoglobin–oxygen saturation (Lumb & Thomas, 2020). Oxygen remaining after aerobic respiration resides in the venous circulation, forming a venous reservoir (Andersen et al., 2018). This acts as a temporal buffer, enabling tissues to manage temporary mismatches between ${\dot{V}}_{O_{2}}$ and delivery. Although often described as *extraction*, oxygen transfer from haemoglobin occurs solely through stepwise diffusion down sequential partial‐pressure gradients. Eventually, when the venous reservoir is depleted, resuscitation of the microcirculation ceases. At this *critical buffering threshold*, intracellular oxygen levels fall abruptly, and the risk of injury escalates.

## 4 CLINICAL PHENOTYPES AND OXYGEN DIFFUSION KINETICS

The retina and brain differ in their vulnerability due to different microvascular structures and metabolic characteristics. In the developing retina, immature peripheral avascular zones are vulnerable to elevated arterial oxygen tension, which inhibits physiological angiogenesis. Later, steep proximal gradients cause intracellular hyperoxia, reducing vascular endothelial growth factor (VEGF) and stopping vessel growth. Later episodes of hypoxia trigger a pathological response of neovascularisation or retinopathy (of prematurity) (Gaynon, 2006).

The immature microvascular structure in the developing periventricular white matter causes wide radial and longitudinal diffusion distances, leading to a shallow capillary–cell gradient and a narrow buffer for aerobic metabolism. Additionally, this region has high metabolic demands, further threatening the safety margin. These features make the tissue vulnerable to the radial extinction gradient, in which small changes in perfusion or oxygen content result in disproportionate decreases in intracellular oxygen tension. Furthermore, when buffering capacity decreases, as with anaemia, hypotension or bradycardia, or when metabolic demands increase, such as during hyperthermia, the tissue is more likely to cross the extinction point. Repeated or prolonged hypoxic episodes then produce the characteristic pattern of periventricular injury, phenotypically recognised as diplegic cerebral palsy (CP) (Table 1).

**TABLE 1:** Phenotypic consequences of variations in constituent parts of oxygen physiology (at STPC) in vulnerable preterm infants.

State	Hb (g/L)	$S_{aO_{2}}$ (%)	Cap‐cell gradient (**Wilson et al.,** 1988)	Distance threshold	Buffering capacity	Dominant physiology	Phenotype
Normoxia	135	92	Normal	Below spatial threshold	Adequate	Balanced	Normal outcome
Hypoxia	135	80	Shallow	Near extinction threshold	Low	Borderline intracellular oxygen	↑ Risk of CP
Hyperoxia	135	100	Steep	Below spatial threshold	Normal	Excess intracellular oxygen proximally	↑ Risk of ROP
Anaemic hypoxia	80	80	Shallow	Spatial + extinction thresholds exceeded	Very low	Catastrophic reduction in flux	↑ death + CP
Anaemic mixed	80	100	Steep	Wider spatial radial threshold	Low	Proximal hyperoxia + distal hypoxia	↑ ROP + ↑ CP

Corresponding approximate physiological values: normoxia: $P_{aO_{2}}$ ∼60 mmHg, $C_{aO_{2}}$ 16.8 mL/dL, cytosolic $P_{O_{2}}$ ∼10 mmHg; hypoxia: $P_{aO_{2}}$ ∼45 mmHg, $C_{aO_{2}}$ 14.6 mL/dL, cytosolic $P_{O_{2}}$ < 10 mmHg. hyperoxia: $P_{aO_{2}}$ > 100 mmHg, $C_{aO_{2}}$ 18.4 mL/dL, cytosolic $P_{O_{2}}$ > 30 mmHg; anaemic hypoxia: $C_{aO_{2}}$ ∼8.7 mL/dL; anaemic mixed: $C_{aO_{2}}$ ∼11 mL/dL, cytosolic $P_{O_{2}}$ > 30 mmHg proximally. Estimate of intracellular oxygen tension, diffusion gradient, spatial threshold, venous buffering capacity, and the resultant physiological or injury phenotype. Abbreviations: $C_{aO_{2}}$, arterial oxygen content; CP, cerebral palsy; Hb, haemoglobin concentration; $P_{aO_{2}}$, arterial oxygen partial pressure; ROP, retinopathy of prematurity; $S_{aO_{2}}$, arterial oxygen saturation.

## 5 ALTERATION OF THE CAPILLARY TO CELL GRADIENT

The NeOProM meta‐analysis of five large, conceptually similar trials comparing low (85–89%) versus high (91–95%) cutaneous oximetry shows how population‐based cutaneous oximetry targets influence clinical outcomes in very preterm infants (Askie et al., 2018). Study oximeters were intentionally offset to maintain blinding, meaning the displayed 88–92% corresponded to different arterial partial pressure of oxygen ($P_{aO_{2}}$) values between groups. Also, the width of the indicative $P_{aO_{2}}$ band increased asymmetrically as saturation targets rose because of the sigmoid shape of the oxyhaemoglobin dissociation curve. For example, at a displayed $S_{pO_{2}}$ of 87%, the indicative $P_{aO_{2}}$ differs by about 6 mmHg (52 mmHg in the low target group versus 58 mmHg in the high). However, this difference widens to 14 mmHg (60 vs. 74 mmHg) at $S_{pO_{2}}$ of 92% (Table 2).

**TABLE 2:** Selected outcomes by assigned saturation target range (Askie et al., 2018).

	Saturation target		
Variable	85–89%	91–94%	
Death	19.9%	17.1%	RD 2.8% [95% CI: 0.6% to 5%]
Treatment of ROP	10.9%	14.9%	RD −4.0% [95% CI: −6.1% to −2.0%]

Lower (85–89%) versus higher (91–94%) targets with corresponding risk differences (RD, 95% CI). Abbreviations: CI, confidence interval; RD, risk difference; ROP, retinopathy of prematurity.

It is important to recognise that these trials manipulated the assigned *population‐based* saturation target, not the *actual* saturation achieved in individual infants. The resulting treatment effect, therefore, represents a population‐wide displacement of the arterial driving pressure gradient, altering population‐level diffusion kinetics rather than reflecting individual physiological exposure.

However, the link between diffusion kinetics and clinical outcomes becomes clear only when data are examined at the individual level. The COT investigators analysed oxygen saturation data from all 1035 infants enrolled in the randomised trial, regardless of the allocated cutaneous saturation target range (Poets et al., 2015). The median monitoring period was 68.3 days per infant. The study primarily examined the cumulative effect of severe desaturation episodes ($S_{pO_{2}}$ < 80% for 60 s or more) on individual outcomes. These episodes were grouped within study gestational age categories. There was a strong dose–response relationship between the frequency, depth and duration of desaturation and all adverse outcomes. Higher cumulative desaturation exposure (high area under the curve values) was associated with increased odds of late death or disability (odds ratio (OR) 2.77; 95% CI: 1.60–4.80) and motor impairment (OR 5.47; 95% CI: 2.58–11.59). Similarly, Di Fiore and colleagues showed that adverse outcomes are more closely associated with individual desaturation burden than with assigned oxygen saturation targets (Di Fiore et al., 2012).

**FIGURE 2:**
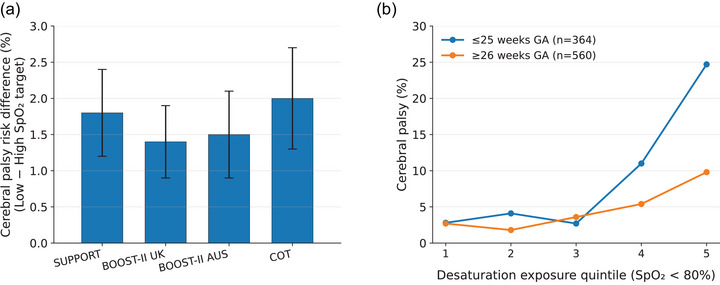
Population‐based oxygen saturation targets versus individual oxygen exposure and cerebral palsy risk. (a) Absolute risk difference in cerebral palsy between low and high oxygen saturation target ranges across randomised trials of oxygen targeting. Bars represent point estimates with 95% confidence intervals, demonstrating modest and overlapping population‐level effects of target allocation. (b) Cerebral palsy prevalence according to quintiles of individual intermittent desaturation exposure ($S_{pO_{2}}$ < 80%), stratified by gestational age. Risk increases progressively with greater desaturation burden, particularly in infants born ≤25 weeks’ gestation, illustrating divergence between population‐level target effects and outcomes associated with individual oxygen physiology (Poets et al., 2015).

For example, data from the reanalysis of the COT trial indicate that the rate of CP among infants without desaturation events was 2.6%. This increased to 5.1% (OR 1.81, 95% CI: 1.01–3.25) in those with desaturation events lasting at least 1 min. When desaturation was accompanied by bradycardia, the risk rose to 8.2% (OR 2.88; 95% CI: 1.55–5.35). The risk increased further in infants with desaturation greater than 1 min (approximately 12%) (Poets et al., 2015) (Figure 2). It is likely that deep desaturation leads to intracellular hypoxia, particularly when the diffusion gradient is shallow, and the event crosses the extinction threshold. Moreover, when desaturation is accompanied by anaemia and/or bradycardia, impaired oxygen delivery may limit microcirculatory resuscitation (buffering capacity), expanding the radius and duration of hypoxic injury.

## 6 THE EFFECT OF FETAL HAEMOGLOBIN ON OXYGEN KINETICS

Haemoglobin morphology adds complexity to oxygen diffusion kinetics in preterm infants. Fetal haemoglobin (HbF) has a higher affinity for oxygen than adult haemoglobin (HbA), facilitating oxygen transfer at the placental interface. After birth, however, this same property impairs the reciprocal diffusion of oxygen from blood to tissue (De Halleux et al., 2002; Delivoria‐Papadopoulos et al., 1976). For any given arterial partial pressure, HbF achieves higher saturation and therefore *tops out* earlier, but the adjacent capillary $P_{O_{2}}$ is correspondingly lower. This narrows the diffusion gradient and reduces oxygen flux to the cell, effectively lowering the critical extinction threshold.

In practice, transfusing allogeneic adult red blood cells further complicates this by creating a mixed, or dimorphic, haemoglobin population. After transfusion, the total amount of HbF remains unchanged, but its proportion decreases. Additionally, blood gas co‐oximeters give a blended *P*_50_ value, even though the infant's blood contains two different haemoglobin types with different oxygen affinity (Franz et al., 2020). As a result, both the measurement and modelling of oxygen kinetics in the preterm circulation may underestimate the heterogeneity of diffusion behaviour within the microvasculature.

## 7 BUFFERING CAPACITY

**FIGURE 3:**
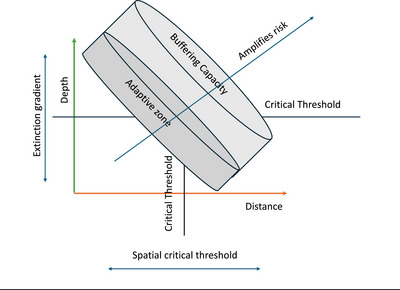
Schematic illustration of how distance (immaturity), depth (desaturation gradient) and duration (systemic buffering) influence the risk of suboptimal intracellular oxygen levels. As the diffusion distance approaches the critical spatial threshold and the desaturation depth nears the extinction gradient, the system's temporal buffering capacity gradually diminishes. Prolonged exposure further depletes venous oxygen reserves, pushing the system beyond its buffering capacity.

Contemporary transfusion practice allows haemoglobin levels to fall from approximately 140 g/L in the first postnatal week to about 96 g/L by week 8 in preterm infant (Kirpalani et al., 2020). During this time, ${\dot{V}}_{O_{2}}$ rises with chronological age, initially driven by thermogenic demand and later by new tissue growth. These changes steadily diminish the arteriovenous oxygen content difference, likely reducing buffering capacity during fluctuations in consumption or delivery (Figure 3).

Centre‐based variation in saturation targets within the effects of transfusion thresholds on neurocognitive outcome (ETTNO) shows an asymmetric interaction between transfusion strategy (haemoglobin) and outcomes (Franz et al., 2025). Infants randomised to restrictive transfusion thresholds in centres with lower saturation targets have higher rates of CP than those in centres with higher saturation targets (5.2% (10 of 191) vs. 3.9% (9 of 229)). Mechanistically, this likely reflects the convergence of the buffering threshold (anaemia) with the extinction threshold (desaturation).

A recent pilot trial in infants with established bronchopulmonary dysplasia before 44 weeks’ post‐menstrual age compared a saturation target of 90–94% with a target of >96% (Bale et al., 2016). However, neither range aligns with the target ranges tested in any of the NeoProM trials, which makes interpretation challenging (Askie et al., 2018). The pilot study defined desaturation similarly to the POET reanalysis of the COT trial (Poets et al., 2015). The higher saturation target inevitably raises arterial and alveolar $P_{O_{2}}$, increasing the capillary–cell gradient and exposing the alveoli to higher oxygen levels. Although fewer hypoxaemic episodes were recorded, this probably reflects oversaturation rather than an improvement in the underlying diffusion kinetics. In fact, the STOP‐ROP trial, albeit in a slightly different population, showed that targeting very high saturations led to higher inspired oxygen concentrations and increased pulmonary morbidity.

## 8 OTHER APPROACHES

Cerebral near‐infrared spectroscopy (NIRS) uses specific light wavelengths to interrogate haemoglobin chromophores in cerebral tissue. The resulting signal represents mixed‐compartment tissue oxygen saturation, typically assumed to comprise ∼75% venous blood (Bale et al., 2016). Since NIRS measures residual oxygen after stepwise diffusion (extraction), it serves as an indicator of tissue oxygen adequacy (Andersen et al., 2017, 2018; Bale et al., 2014).

The Safeguarding the Brain of Our Smallest Infants Phase (SafeBoosc) (Hansen et al., 2023; Pellicer et al., 2013; Plomgaard et al., 2016) and Cerebral Oxygen Saturation to Guide Oxygen Delivery (COSGOD) (Pellicer et al., 2013, Pichler et al., 2023 and Plomgaard et al., 2016) trials established the clinical feasibility of NIRS‐guided oxygen management in preterm infants. By targeting cerebral tissue oxygenation in real time, these studies demonstrated that tissue‐based oximetry can be safely integrated into routine care both during resuscitation and after admission. Conceptually, arterial oximetry and cerebral NIRS provide information at opposite points in the oxygen cascade.

In both SafeBoosC III and COSGOD III, intervention algorithms mainly adjusted inspired oxygen concentration to modify the arterial to (venous) tissue diffusion gradient (Pichler et al., 2023; Stafford et al., 2023). This strategy optimises the radial capillary‐to‐cell oxygen gradient but does not directly influence the structural aspects (microvascular geometry and maturity) or the temporal characteristics (buffering capacity). In SafeBoosC III, NIRS‐guided titration reduced the burden of cerebral hypoxia (low tissue oxygen saturation) without affecting mortality or severe disability at 2 years. Meanwhile, COSGOD III reported a 4.3% increase (not statistically significant) in survival without cerebral injury. Longer‐duration or differently timed interventions might have different effects, particularly considering COT reanalyses that identify later injury windows (Poets et al., 2015).

Collectively, these trials clarify the current scope of gradient‐based optimisation. Yet systemic oxygen kinetics may remain limited when buffering capacity and diffusion gradients are jointly constrained. A logical next step is to evaluate interventions that combine cerebral tissue oxygenation monitoring with strategies that address both haemoglobin‐dependent buffering capacity and the capillary‐to‐cell diffusion gradient.

## 9 A LOOK TO THE FUTURE

There is increasing interest in the automated regulation of inspired oxygen concentration in preterm infants receiving supplemental oxygen (Stafford et al., 2023). These systems adjust the inspired oxygen to keep the infants within predefined saturation limits, acting on the arterial compartment and thereby modifying the gradient (Van Zanten et al., 2017). They do not affect systemic buffering capacity, which is especially important during deep, recurrent, or prolonged desaturation when venous oxygen reserves support the recovery of the microcirculation.

The mitochondrial redox state provides greater insight into intracellular oxygen sufficiency, especially when delivery and consumption appear adequate, but mitochondrial function remains impaired. Cytochrome *c* oxidase (CCO), the final enzyme of the electron transport chain, catalyses the reduction of molecular oxygen to water and acts as a sensitive marker of cellular oxygen tension. Broadband and multiwavelength NIRS techniques measure oxidised cytochrome *c* oxidase (oxCCO) as an indicator of mitochondrial redox balance, reflecting the local equilibrium between oxygen supply and cellular utilisation (Bale et al., 2014; Cooper & Springett, 1997; Wilson et al., 1988). Although challenges persist in spatial resolution and signal interpretation, oxCCO is the closest non‐invasive marker of downstream metabolic adequacy.

The progression from pulse oximetry through tissue oximetry to mitochondrial redox monitoring illustrates a step‐by‐step movement along the oxygen diffusion pathway, from arterial supply to tissue delivery and cellular use. Each stage provides information closer to the site of injury. However, each step also increases the complexity of interpreting signals and reduces spatial resolution. The challenge is integrating these complementary measurements into a clear model that considers spatial thresholds (immaturity), gradient dynamics (arterial $P_{O_{2}}$) and buffering capacity (haemoglobin and cardiac output).

## 10 CONCLUSION

Although most neonatal clinicians consider the assessment of supplemental oxygen exposure to be well established, it remains a significant unresolved issue in our discipline. Saturation target ranges are typically set at an institutional level, meaning that individual clinicians may not know the rationale for the chosen limits and parents are rarely informed of their implications. Yet this binary choice effectively shifts infants along the oxygen diffusion–injury continuum, trading the risk of one phenotype for another.

The three determinants of distance (immaturity), depth (gradient) and duration (buffering) each have dynamic thresholds that interact to shape intracellular oxygen tension. These thresholds vary over time and may differ between tissues even within the same infant. Addressing this complexity requires tools that directly assess intracellular oxygen sufficiency. Measurement of mitochondrial redox state, specifically CCO oxidation, using broadband or multiwavelength NIRS provides the closest non‐invasive insight into downstream metabolic adequacy.

Administering oxygen to preterm infants must therefore account for the combined effects of diffusion kinetics and metabolic demand, in which the spatial, extinction and buffering thresholds jointly determine vulnerability to hyperoxic or hypoxic injury. Future research should integrate these principles into a single mechanistic model that accounts for rising metabolic needs and evaluates interventions that modify both the diffusion gradient and the venous oxygen buffer.

## AUTHOR CONTRIBUTIONS

Chad Andersen conceived and designed the manuscript and wrote the primary draft. Danielle Bailey, Tara Crawford, and Michael Stark provided critical revisions in addition to adapting figures and Tables. Authors declare that all the intellectual property is their own. All authors have read and approved the final version of this manuscript and agree to be accountable for all aspects of the work in ensuring that questions related to the accuracy or integrity of any part of the work are appropriately investigated and resolved. All persons designated as authors qualify for authorship, and all those who qualify for authorship are listed.

## CONFLICT OF INTEREST

None declared.

## FUNDING INFORMATION

This research (review) manuscript has received no specific grant from any funding agency in the public, commercial, or not‐for‐profit sectors.
